# Multilineage Potential of Stable Human Mesenchymal Stem Cell Line Derived from Fetal Marrow

**DOI:** 10.1371/journal.pone.0001272

**Published:** 2007-12-05

**Authors:** Atsushi Nagai, Woo K. Kim, Hong J. Lee, Han S. Jeong, Kwang S. Kim, Seok H. Hong, In H. Park, Seung U. Kim

**Affiliations:** 1 Division of Neurology, Department of Medicine, University of British Columbia, Vancouver, Canada; 2 Department of Laboratory Medicine, Shimane University School of Medicine, Izumo, Japan; 3 Institute for Regnerative Medicine, Gachon University Gil Hospital, Inchon, Korea; 4 Department of Physiology, Chonnam National University Medical School, Gwangju, Korea; University of Sydney, Australia

## Abstract

Human bone marrow contains two major cell types, hematopoietic stem cells (HSCs) and mesenchymal stem cells (MSCs). MSCs possess self-renewal capacity and pluripotency defined by their ability to differentiate into osteoblasts, chondrocytes, adipocytes and muscle cells. MSCs are also known to differentiate into neurons and glial cells *in vitro*, and *in vivo* following transplantation into the brain of animal models of neurological disorders including ischemia and intracerebral hemorrhage (ICH) stroke. In order to obtain sufficient number and homogeneous population of human MSCs, we have clonally isolated permanent and stable human MSC lines by transfecting primary cell cultures of fetal human bone marrow MSCs with a retroviral vector encoding v-*myc* gene. One of the cell lines, HM3.B10 (B10), was found to differentiate into neural cell types including neural stem cells, neurons, astrocytes and oligodendrocytes *in vitro* as shown by expression of genetic markers for neural stem cells (nestin and Musashi1), neurons (neurofilament protein, synapsin and MAP2), astrocytes (glial fibrillary acidic protein, GFAP) and oligodendrocytes (myelin basic protein, MBP) as determined by RT-PCR assay. In addition, B10 cells were found to differentiate into neural cell types as shown by immunocytochical demonstration of nestin (for neural stem cells), neurofilament protein and β-tubulin III (neurons) GFAP (astrocytes), and galactocerebroside (oligodendrocytes). Following brain transplantation in mouse ICH stroke model, B10 human MSCs integrate into host brain, survive, differentiate into neurons and astrocytes and induce behavioral improvement in the ICH animals. B10 human MSC cell line is not only a useful tool for the studies of organogenesis and specifically for the neurogenesis, but also provides a valuable source of cells for cell therapy studies in animal models of stroke and other neurological disorders.

## Introduction

Human bone marrow contains two major cell types, hematopoietic stem cells (HSCs) and mesenchymal stem cells (MSCs). MSCs possess self-renewal capacity and pluripotency defined by their ability to differentiate into bone, fat, cartilage and muscle [1]–[4]. MSCs are also known to differentiate into neurons and glial cells *in vitro* and *in vivo* [5]–[7].

Two major types of stroke are ischemic stroke and intracerebral hemorrhage (ICH), and ICH represents at least 15% of all strokes in the western population [8], while in Asia including China, Japan and Korea ICH occupies considerably higher proportion at 50–60%[9]. ICH is a lethal stroke type, as mortality approaches 50% and neurological disability in survivors is common. Since medical therapy against ICH such as mechanical removal of hematoma, prevention of edema formation by drugs, and reduction of intracranial pressure, shows only limited effectiveness, alternative approach is required [10], [11].

Previous studies have reported that MSCs engrafted in animal models of stroke survive and ameliorate neurological deficits in the animals [12]–[15], raising the possibility of therapeutic potential of MSCs for repair of damaged brain in ICH animal models and patients. However, the studies related to the cellular and molecular properties of human MSCs run into difficulty in obtaining sufficient number and homogeneous population of human MSCs, and primary MSCs can be provided for only a limited time before they undergo senescence. Generation of sustainable human MSC clones is necessary to circumvent these problems. Previously we have isolated clonal human neural stem cell lines that had been immortalized by a retroviral vector encoding v-*myc* oncogene[16]–[19], and these cells show multipotent differentiation capacity to differentiate into neurons and glial cells [16]–[18], ameliorate neurological deficits in animal models of stroke [20]–[24], Parkinson disease [25], Huntington disease [26], [27] and lysosomal storage disease [28] following their transplantation into the brain. Using a similar procedure, we have generated clonal immortalized human mesenchymal stem cell lines by transfecting primary cell cultures of fetal human bone marrow mesenchymal stem cells with a retroviral vector encoding v-myc oncogene. One of the cell lines, HM3.B10 (B10), was found to differentiate into glial cells *in vitro* and *in vivo*, and also restore functional deficits in mice with experimenal ICH following brain transplantation.

## Methods

### Primary culture of human bone marrow cells

Bone marrow cells (MSCs) were obtained from human fetal spinal vertebrae of 12–15 weeks' gestation. The permission to use embryonic tissues was granted by the Clinical screening committee for research involving human subjects of the University of British Columbia. Bone marrow tissues were isolated from the vertebrae using two scapels and grown in 60 mm culture dishes with Modified Eagle medium alpha (MEM-α) supplemented with 10% fetal bovine serum and 25 µg/ml gentamicin (feeding medium). After 2–3 weeks, cultures reached confluency and MSCs were passaged further. MSCs used in the present study were cultures at 4th to 11th passage.

### Retrovirus-mediated gene transfer

**Figure 1:**
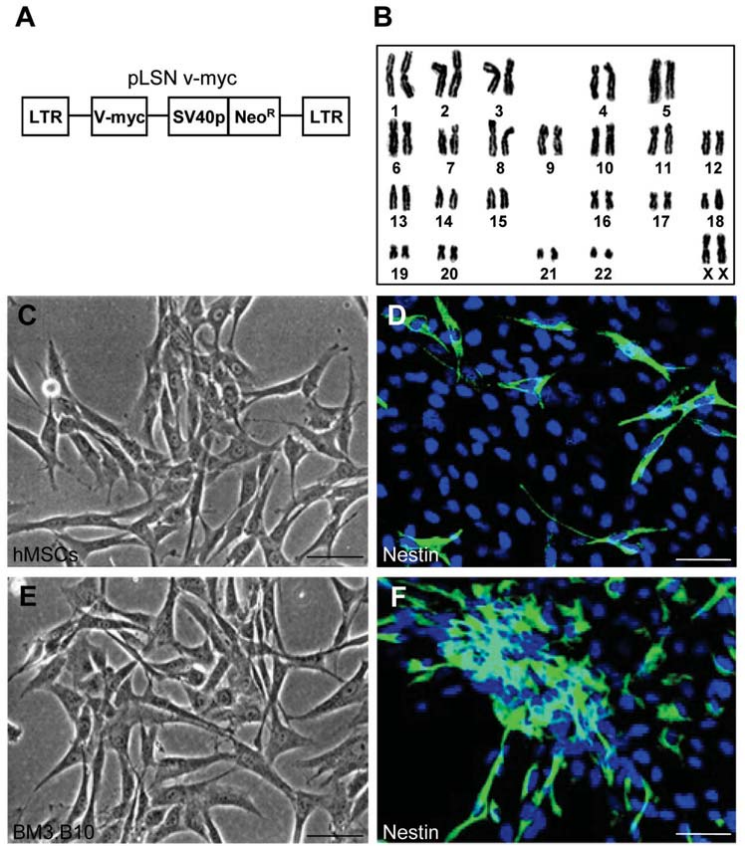
Generation of HM3.B10 (B10) immortalized human bone marrow mesenchymal stem cell (MSC) line. (A): The retroviral vector encoding v-myc used for the generation of B10 immortalized human MSC line from primary culture of human fetal bone marrow cells. The vector has an internal SV40 promoter (SV40) from which the neomycin-resistant gene (NeoR) is transcribed and translated. The myc oncogene (v-myc) is transcribed from the LTR (long terminal repeat). (B): Karyotyping analysis of B10 cells revealed the normal human karyotype of 46, XX. (C): Phase contrast microscopy of primary human MSCs derived from fetal human bone marrow (16 weeks gestation). Bar indicates 20 µm. (D): Immunofluorescence microscopy of primary human MSCs expressing nestin staining, a cell type specific-marker for neural stem cells. Cell nuclei are labeled by DAPI. (E): Phase contrast microscopy of B10 human MSC cell line at passage 11. (F): Immunofluorescence microscopy of B10 cells expressing nestin staining. Bars in (C–F) indicate 20 µm.

Human MSCs were subjected to retrovirus mediated-transduction of v-myc by LSNv-myc construct and subsequent cloning. An amphotropic replication-incompetent retroviral vector encoding v-myc oncogene (transcribed from mouse leukemia virus LTR plus neomycin-resistant gene transcribed from a SV40 early promotor) was used (Figure 1A). This amphotropic vector, LSNv-myc, was generated in our laboratory using the ecotropic retroviral vector encoding v-myc (American Type Culture Collection, Manhasset, VA) to infect PA317 amphotropic packaging cell line. Infection of human MSCs in 6 well plates was performed twice by the established procedures [17], [19], [29]. Briefly, 2 ml of supernatant (4×10^5^ CFUs) from the PASK packaging cell line and 8 µg/ml polybrene (Sigma) were added to target cells in 6 well plates and incubated for 4 hr at 37°C. The medium was then replaced with fresh growth medium. Infection was repeated 24 hr later. Seventy-two hr after the second infection, infected cells were selected with G418 (250 µg/ml, Sigma) for 7–14 days and large clusters of clonally derived cells were individually isolated and grown in 6 well plates. Individual clones were generated by limited dilution and propagated further. At this phase of isolation, individual clones were designated as HM3 human MSC cell lines. One of these clones, HM3.B10 (B10), was subjected to further study.

### Fluorescence activated cell sorting

For fluorescence activated cell sorter (FACS), B10 cells were detached by a brief trypsin treatment and stained sequentially with immunofluorescence conjugated antibodies, fixed with 2% paraformaldehyde for 5 min, and then analyzed with flow cytometer (FACS Vantage, Becton Dickinson). The antibodies utilized for FACS analysis are shown in Table 1.

**Table 1:** Phenotypic analysis of B10 cells with FACS.

Epitope	Source of Ab	HM3.B10
CD13	Immunotechnology	(+)
CD29 (β1-integrin)	Becton Dikinson	(+)
CD44 (endoglin)	Becton Dikinson	(+)
CD49b	Fujisawa	(+)
CD90 (Thy-1)	Becton Dikinson	(+)
CD166	Becton Dikinson	(+)
CD14	Becton Dikinson	(−)
CD34	Becton Dikinson	(−)
CD45	Becton Dikinson	(−)
HLA-DR	Immunotechnology	(−)

### Differentiation culture conditions

Adipogenic differentiation of B10 cells was induced by growing the cells in a 6-well plate in DMEM containing 10% FBS, 5 µg/ml insulin, 1 µM dexamethasone, 100 nM indomethacin and 0.5 mM methylisobutylxanthine (Sigma) for 48 h, then cells were incubated in the same medium without dexamethasone. One week after the induction, cells were stained with oil-red. To induce osteogenic differentiation, B10 cells were cultured in DMEM containing 10 mM β-glycerophosphate, 0.2 mM ascrobate-2-phosphate, 10 nM dexamethasone. After 14 days, osteogenic differentiation was evaluated by alkaline phosphatase staining. For chondrogenic differentiation, pellets of B10 cell were cultured for 3 weeks in the presence of transforming growth factor-β1 (TGFβ-1) in DM4 serum-free medium (DMEM containing 10 µg/ml human insulin, 10 µg/ml human transferrin, 3 nM sodium selenite, 5 nM hydrocortisone and 100 pM triiodothyronine) [30]. This medium was replaced every 3–4 days for 21 days. Development of chondrogenic differentiation was determined by staining the pellet with Alcian blue. For neuronal induction, B10 cells were grown in DMEM containing 1% FBS and supplementary 100 ng/ml bFGF for 14 days. Induction of neuronal and glial phenotypes in B10 cells was confirmed by immunostaining with antibodies specific for neurofilament-H, β-tubulin III and glial fibrillary acidic protein (GFAP). In order to induce neuronal and glial phenotypes in B10 cells, feeding media containing 1% FBS and various growth factors (bFGF, EGF, VEGF, NGF, BDNF and CNTF) or modulators (phorbol ester and forskolin) were tested, and bFGF at 100 ng/ml proved to be most effective in inducing neuronal phenotype.

### Immunocytochemistry

Immunochemical determination of cell type specific markers in B10 cells was performed as follows: B10 cells were grown on poly-D-lysine-coated Aclar plastic coverslips (9 mm in diameter) for 3–14 days, fixed in cold methanol for 10 min at −20°C, air dried, and incubated with primary antibodies, followed by biotinylated secondary antibodies and avidin-biotin complex (ABC, Vector, Burlingame, CA) and visualized with 3-amino-9-ethyl carbazole (Sigma) chromogen development [29], [31]. For immunofluorescence studies, cultures were incubated with primary antibodies, followed by fluorescent secondary antibody (Alexa Fluor 488 anti-mouse IgG (1∶200, Molecular Probe, Eugene, OR) or Alexa Fluor 594 anti-rabbit IgG (1∶200) for 1 hr at RT and viewed under an Olympus laser confocal microscope. Cell type-specific markers used were neurofilament-L (NF-L), neurofilament-M (NF-M) and tubulin-β isotype III (β-tubulin III) for neurons, glial fibrillary acidic protein (GFAP) for astrocytes and cyclic nucleotide phosphodiestelrase (CNPase) for oligodendrocytes. Mouse monoclonal antibodies specific for NF-L, NF-M and tubulin-β III, and CNPase were obtained from Chemicon (Temecula, CA), and rabbit anti-GFAP antibody was obtained from DAKO (Carpinteria, CA). For oil-red staining for adipose cells, cells were fixed with 4% paraformaldehyde for 10 min, washed with PBS, incubated with Oil-Red-O (Sigma) for 10 min and counterstained with Mayer hematoxylin for 3 min. For alkaline phosphatase staining for osteoblasts, cells were fixed with 4% paraformaldehyde for 10 min, washed with PBS, incubated with fast 5-bromo-4-chloro-3-indolyl phosphate and nitroblue tetrazolium substrate (Sigma) for 20 min. For Alcian blue staining for chondroblasts, cell pellets were fixed with 4% paraformaldehyde at 4°C for 2 h, embedded in paraffin and sections stained with 0.5% Alcian blue solution for 15 min and counterstained with Myer hematoxylin for 1 min.

### RT-PCR analysis

RT-PCR was performed with oligonucleotide primers in Table 2. Sense and antisense primers of each primer pair were set at a different exon, respectively, to avoid DNA contamination. Total RNA was extracted using TRIzol reagent (GIBCO-BRL, Gaithersburg, MD). Complimentary DNA (cDNA) templates from each sample were prepared from 1 µg of total RNA primed with oligo dT primers (Pharmacia, Gaithersburg, MD) using 400 units of MMLV reverse transcriptase (Promega, Madison, WI) followed by 30 PCR amplification cycles (94°C for 30 seconds, annealing at 60°C for 60 seconds, and extension at 72°C for 90 seconds). Glyceraldehyde-3-phosphate dehydrogenase (GAPDH) was used as a reaction standard. Ten µL of each PCR product was analyzed by 1.5% agarose gel electrophoresis. Authentic bands were determined by selective enzyme digestion.

**Table 2:** Sequence of PCR Primers.

Gene	sense	antisense
Lipoprotein lipase	CTTCTGTTCTAGGGAGAAAGTG	TGCTGTGTAGATGAGTCTGATT
Osteopontin	GAAGGACAGTTATGAAACGAGT	AACATAGACATAACCCTGAAGC
Osteocalcin	ATGAGAGCCCTCACACTCCT	CAAGGGGAAGAGGAAAGAAG
PTH receptor	AACTACTACTGGATTCTGGTGG	CTCCAAGATTTCTTGATCTCAG
Syndecan	CCTTCACACTCCCCACAC	GGCATAGAATTCCTCCTGTTG
Perlecan	CATAGAGACCGTCACAGCAAG	ATGAACACCACACTGACAACC
Collagen type II	ACGGCGAGAAGGGAGAAGTTG	GGGGGTCCAGGGTTGCCATTG
Oct-4	AGGAGATATGCAAAGCAGAA	AGAGTGGTGACGGAGACAG
Mash1	CCAACTACTCCAACGACTTG	GAAAGCACTAAAGATGCAGG
Otx-2	ATGCAGAGGTCCTATCCCATG	CTTATAATCCAAGCAATCAGT
ABCG2	CAAAAACTTGCTGGGTAATC	ACAGAAACCACACTCTGACC
Nestin	CTCTGACCTGTCAGAAGAAT	GACGCTGACACTTACAGAAT
Pax-3	GCCAATCAACTGATGGCTTT	CATTCGAAGGAATGGTGCTT
Pax-6	GGTCTGTACCAACGATAACATAC	CTGATAGGAATATGACTAGGTGTG
Wnt-1	ATGGGGCTCGCTGTT	CCCACTCATGCAGGA
NeuroD1	CCGACAGAGCCCAGATGTAGTTCTT	GCCCCAGGGTTATGAGACTATCACT
Sox-1	AACCCCAAGATGCACAACTC	TAGCCCAGCCGTTGACAT
Sox-2	AGTACAACTCCATGACCAGC	TTACTCTCCTCTTTTGCACC
Sox-3	AGAACCCCAAGATGCACAAC	CTGCACGAGCGAGTAGGC
Sox-9	TGAAGAAGGAGAGCGAGGAA	GGGGCTGGTACTTGTAATCG
Sox-10	AGGAGAAGGAGGTTGACTGT	TCCTCAAAGCTACTCTCAGC
NF-L	TCCTACTACACCAGCCATGT	TCCCCAGCACCTTCAACTTT
NF-M	TGGGAAATGGCTCGTCATTT	CTTCATGGAAGCGGCCAATT
NF-H	CTGGACGCTGAGCTGAGGAA	CAGTCACTTCTTCAGTCACT
Synaptophysin	CTTCCTGCAGAACAAGTACC	CTTAAACACGAACCACAGGT
MAP2	CTCAACAGTTCTATCTCTTCTTCA	TCTTCTTGTTTAAAATCCTAACCT
MBP	ACACGGGCATCCTTGACTCCATCGG	TCCGGAACCAGGTGGGTTTTCAGCG
GFAP	GCAGAGATGATGGAGCTCAATGACC	GTTTCATCCTGGAGCTTCTGCCTCA
NGF	TCATCATCCCATCCCATCTTCCAC	CACAGCCTTCCTGCTGAGCACAC
BDNF	ATGACCATCCTTTTCCTTACT	CTATCTTCCCCTTTTAATGGT
NT3	ATGTCCATCTTGTTTTATGTGA	TCATGTTCTTCCGATTTTTC
GDNF	ATGAAGTTATGGGATGTCGT	TTAGCGGAATGCTTTCTTAG
CNTF	ATGGCTTTCACAGAGCATT	AACTGCTACATTTTCTTGTTGTT
bFGF	GGGTGGAGATGTAGAAGATG	TTTATACTGCCCAGTTCGTT
IGF-1	AAATCAGCAGTCTTCCAACCCA	CTTCTGGGTCTTGGGCATGT
HGF	AGGAGAAGGCTACAGGGGCAC	TTTTTGCCATTCCCACGATAA
VEGF	GAAGTGGTGAAGTTCATGGATGTC	CGATCGTTCTGTATCAGTCTTTCC
TGFβ-1	ACCTGCAAGACTATCGACAT	TAGTACACGATGGGCAGC
c-kit	TATACAACCCTGGCATTATGTCC	TGCGAAGGAGGCTAAACCTA
GAPDH	CATGACCACAGTCCATGCCATCACT	TGAGGTCCACCACCCTGTTGCTGTA

### Electrophysiology

B10 cells grown on coverslips for 11–14 days were placed in a recording chamber on the stage of an inverted microscope (Eclipse TE2000-S, Nikon, Tokyo, Japan), and membrane currents were recorded using the whole-cell patch clamp technique [16]. Patch micropipettes having resistance of 2–4 MΩ were pulled by a puller (P87, Sutter Instruments, Novato, CA) from borosilicate glass capillaries (G150T-3, Warner Instruments, CT) and fire-polished using a microgorge (MF-79, Narishige, Japan). The pipette were filled with an intracellular-like solution containing (in mM) 140 KCl, 5 NaCl, 1 CaCl_2_, 10 HEPES, 5 EGTA, 2 Mg-ATP for the inward Na^+^ currents or outward K^+^ currents measurement. The pH was adjusted to 7.3 with KOH and filtered before use. The standard external solution was comprised of (in mM) 140 NaCl, 5 KCl, 1 CaCl_2_, 1 MgCl_2_, 10 glucose, 10 HEPES. The solutions were adjusted to pH 7.3 with NaOH. Whole-cell currents were recorded at 22–24°C using a patch-clamp amplifier (Axopatch 200B, Axon Instruments, Foster City, CA) and digitized by a analog-to-digital interface (Digidata 1320, Axon Instruments). Membrane currents were low-pass filtered at 2 kHz and sampled at 50 kHz, then stored on the hard disk of an IBM-compatible computer using pClamp8.2 (Axon Instruments). For voltage-clamp measurements, B10 cells were held at −80 mV and depolarized in 10 mV steps between −70 and +40 mV. The neurotransmitters used were GABA (1 mM), glycine (100 µM), and N-methyl-D-aspartate (100 µM), and the inhibitors of neurotransmitters were bicuculline (10 µM), strychnine (1 µM), and a mix of APV (2 µM) and CNQX (6-cyano-7-nitroquinoxaline-2,3-dione; 2 µM) respectively. The bathing solution or the drug-containing solution was applied to the recording chamber via a gravity-fed perfusion system.

### Mouse ICH model

Male ICR mice weighing 20 to 30 g (7 weeks) were used for ICH model as described previously [21]–[24]. Mice were anesthetized with intraperitoneal injections of 1% ketamine (60 mg/kg) and placed in stereotactic apparatus. A midline incision was made through the scalp to expose the skull and an injection was made sterotaxically into the striatum with a 10 µl Hamilton syringe at the following coordinates: 0.1 mm anterior and 2.0 mm right lateral to the bregma and 4.0 mm ventral to the cortical surface. Core animal temperature was maintained at 37°C during this time. The control group (n = 5) received a 0.5 µl injection of saline alone and the experimental group (n = 14) received a 0.5 µl injection of saline with 0.075 U of collagenase (type VII, Sigma). The infusion rate for both groups was 0.5 µl/min seconds and once the infusion was complete, the syringe was left in place for 5 minutes. The mice were allowed to recover from surgery in a warm environment for 3 hours. All animal experimental procedures were approved by the Gachon University Animal Care Committee.

### Brain transplantation

In order to detect the grafted cells in host brain, donor cells were infected by adenovirus mediated LacZ gene *in vitro* at 100 MOI (PU/cell) before 24 hr transplantation. Experimental groups are group 1 (control): injection of PBS (2 µl, n = 4); group 2: transplantation of primary MSCs (2×10^5^/2 µl, n = 7); and group 3: transplantation of B10 cells (2×10^5^/2 µl, n = 7). At 7 days after ICH, 2×10^5^ cells (primary human MSCs or B10 cells) in a total fluid volume of 2 µl were transplanted into ipsillateral striatum, 2 mm cranial to the hemorrhagic lesion, calculated from bregma: 0.1 mm anterior and 2.0 mm right lateral to the bregma and 2.0 mm ventral to the cortical surface.

### Behavioral test

Motor function was determined using a rotarod test. In this procedure, animals were placed on the center of rotating axle and the time period the animal remained on the axle was measured. The speed was slowly increased from 4 to 40 rpm with in a period of 2 min 30 seconds. The animals were trained 1 week before administration of collagenase and daily, for a period of 7 days, thereafter.

### Histological examination

Two and six weeks following brain transplantation, the animals were anestherized and perfused with heparinized saline followed by 4% prarformaldehyde in 0.1 M phosphate buffer (pH 7.4). Three sections through the needle entry site, 1.0 mm anterior and 1.0 mm posterior to plane were Nissl stained to analyze the hemisphere area. The total hemispheric areas of each section were traced and measured with an image analysis system (Image-Pro Plus, Media Cybernetics, Silver Spring, MD). The morphometric analyses involved computer-assisted hand delineation of the area of the striatum, cerebral cortex, and ventricle, as well as the whole hemisphere. Serial coronal sections (30 µm) throughout the striatum were cut on a cryostat. β-galactosidase (β-gal) protein expression was detected in grafted MSCs in vivo, incubating in enzymatic X-gal solution (5 mM potassium ferricyanide, 5 mM potassium ferrocyanide, 40 mg/mL X-gal in dimethylformamide) for 4 hr at 37°C. All chemicals were purchased from Sigma. The differentiation of grafted MSCs into neural cells was determined by double-labeling immunofluorescence microscopy. Free-floating sections were briefly quenched with 3% H_2_O_2_ in PBS for 10 min. Sections were incubated in Tris-buffered saline (TBS) containing 5% normal goat serum and 0.3% Triton X-100 for 30 min at RT and then incubated overnight with the following primary antibody mixtures; anti-β-galactosidase (β-gal, 1∶300, Sigma)/anti-tubulin-β isotype III (1∶500, Sigma), anti- β-gal/anti-neurofilament (NF, 1∶100, Zymed) and anti- β-gal/anti-glial fibrillary acidic protein (GFAP, 1∶500, DAKO). Sections were rinsed with TBS and incubated with the fluorescent secondary antibodies mixture of Alexa Fluor 488 anti-mouse IgM (1∶200, Molecular Probe), Alexa Fluor 594 anti-mouse IgG (1∶200) and Alexa Fluor 594 anti-rabbit IgG (1∶200) for 1 hr at RT.

### ELISA assay for in vitro production of human NGF and BDNF

To examine in vitro levels of NGF and BDNF production, primary MSCs and B10 cells were grown in serum-containing medium (10% FBS) for 48 hr. The spent media were collected and stored at −80°C. MSCs and B10 cells were harvested after a brief incubation in PBS containing 0.25% trypsin and 1 mM EDTA at 37°C for 4 min. The cells were lysed in RIPA buffer (150 mM NaCl, 1% Nonidet P-40, 0.5% deoxycholic acid, 0.1% SDS, 50 mM Tris, pH 8.0) containing protease inhibitors, centrifuged (10,000g) for 10 min, and the supernatants were used for the assay. Protein concentrations were determined with Bradford protein assay (Biorad) with bovine serum albumin as a standard [32]. Production of NGF and BDNF in culture supernatants and cell lysates was determined by using ELISA kits specific for human β-NGF (R&D Systems, Minneapolis, MN) or human BDNF (Promega, Madison, WI).

### BDNF ELISA assay in brain sections

Production of NGF and BDNF in culture supernatants and brain homogenates was determined by using ELISA kits specific for human α-NGF (R&D Systems) or human BDNF (Promega). Experimental rats, three groups of 7 each (total n = 21) at 2, 4 and 6 weeks post-transplantation, were anesthetized, decapitated and the brains were removed. Brains were cut in 2 mm coronal sections. Two-millimeter diameter punches centered on each side (ipsilateral and contralateral of grafts) of the neostriatum and adjoining cortex were taken according to the rat brain atlas. Seven samples were collected from both the ipsilateral and contralateral sides. The collected samples were homogenized with lysis buffer (137 mM NaCl, 20 mM Tris, pH 8.0, 1% Nonidet P-40, 10% glycerol, 1 mM PMSF, 10 mg/ml aprotinin, 1 mg/ml leupeptin and 0.5 mM sodium vanadate), centrifuged (14,000g) for 30 min and the supernatants stored at at −70°C until use. Protein concentrations were determined with Bradford protein assay (Biorad) with bovine serum albumin as a standard [32]. Levels of BDNF in tissue lysates were determined by using an ELISA kit specific for human BDNF.

### Stereological cell counts

Total number of LacZ-positve B10 cells in the brain sections from ICH animals was determined by stereological estimation. The sections used for counting covered the entire striatum with hemorrhage lesion and overlying cortex. This generally yielded six or seven sections in a series. Sampling was done using the Computer-assisted stereological toolbox system (Olympus Denmark A/S, Ballerup, Denmark), using an Olympus BX51 microscope, a motorized microscope stage (Prior Scientific, Rockland, MA) run by an IBM-compatible computer, and a microcator (ND 281B, Heidenhain, Schaumburg, IL) connected to the stage and feeding the computer with the distance information in the z-axis. The counting areas were delineated ata 1.25× objective and generatedcounting areas of 150×150 µm. A counting frame (1612 µm^2^) was placed randomly on the first counting area and systemically moved through all counting areas until the entire delineated area was sampled. Actual counting was performed using a 100× oil objective. Guard volumes (4 µm from thetop and 4.6 µm from the bottom of the section) were excluded from both surfaces to avoid the problem of lost caps, and only the profiles that came into focus within the counting volume (witha depth of 10 µm) were counted. The estimate of the total number of Human nuclear antigen (HuNu)-positive B10 cells was calculated according to the optical fractionator formula [33].

### Statistical analysis

Data are presented as means±SEM. The statistical significance between group comparisons for morphological and behavioral data was determined by one-way ANOVA test. P values <0.05 were considered to be statistically significant.

## Results

### Generation of immortalized human mesenchymal stem cell lines

Primary MSCs derived from human fetal bone marrow were transfected with a retroviral vector encoding v-myc oncogene (Figure 1A), and clones of immortalized human bone marrow MSCs were generated (see Methods). Several stable clones of immortalized MSCs were isolated after two rounds of cloning and were expanded further. Twelve human MSC cell lines were isolated and one of the clones, HM3.B10 (B10) was used in the further analysis. B10 cells expressed v-myc oncogene as determined by RT-PCR and carried 46, XX normal human karyotype as shown by a cytogenetical analysis (Figure 1B). They are adherent fibroblast-like cells morphologically similar to primary human MSCs (Figures 1C and D). More than 65% of B10 cells are nestin-positive compared to 12% in primary human MSCs. Doubling time of B10 cells was determined to be 36 hr.

### FACS analysis and pluripotent capability of B10 cells

**Figure 2:**
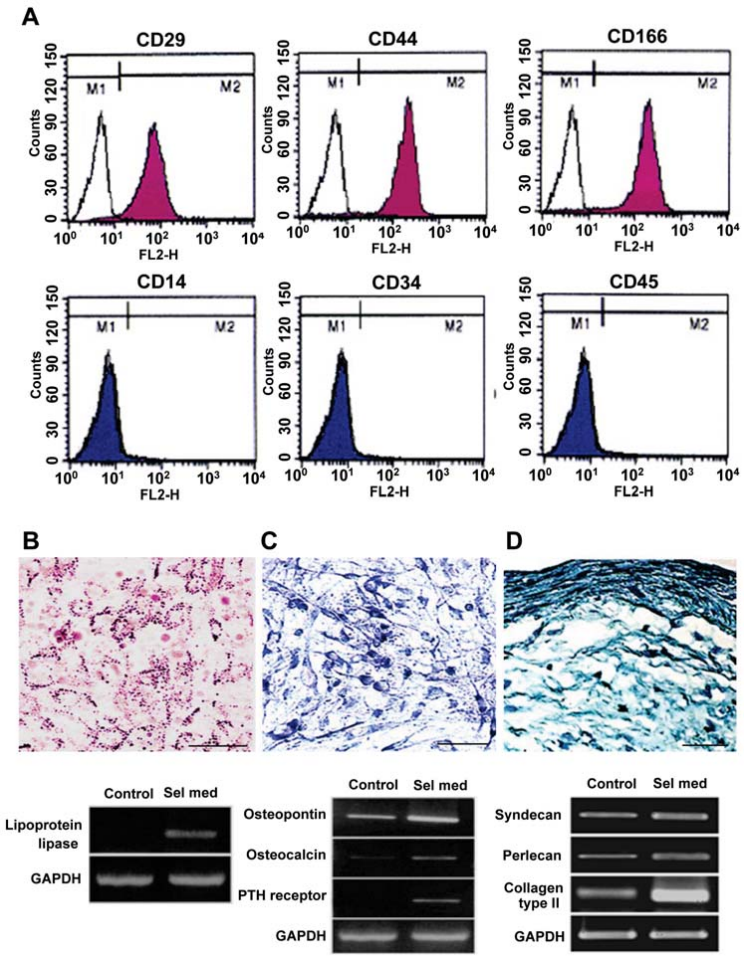
Charactrization and Differentiation of B10 cells carrying phenotypes of human MSCs. (A): B10 cells were labeled with FITC-coupled antibodies specific for CD24, CD29, CD34, CD44, CD45, CD166 or immunoglobulin isotype control antibodies. Surface phenotype was analysed by FACS. Open histograms are for control immunoglobulins and colored histograms are for specific antibodies. MSC markers, CD29, CD44 and CD166 are expressed in B10 cells while hematopoietic stem cell markers, CD14, CD34 and CD45 are not. (B)–(D): B10 cells were cultured for 2 weeks in the selection media specifically designed for adipogenic, osteogenic or chondrogenic differentiation. (B), adipogenic differentiation is shown with oil red O staining and RT-PCR analysis for lipoprotein lipase; (C): Osteogenic differentiation is shown with alkaline phosphatase staining and RT-PCR analysis for osteopontin, osteocalcin and PTH receptor; (D): Chondrogenic differentiation is detected with alcian blue staining and RT-PCR analysis for syndecan, perlecan and type II collagen. Bars indicate 50 µm.

FACS analysis of B10 cells demonstrated that more than 95% of the cells express MSC-specific cell type markers including CD13, CD29 (integrin-β1), CD44 (endoglin), CD49b, CD90 (Thy-1) and CD166, but did not express cell type-specific markers for hematopoietic stem cell (HSC) including CD14, CD34, CD45 and HLA-DR (Figure 2A and Table 1). The phenotypes remained unchanged for more than 40 cell doublings.

To determine whether B10 cells are pluripotent and able to differentiate into various cell types in vitro, B10 cells were cultured in selection media as previously described for human embryonic stem cells [34]. After 7 days of culture in adipogenic culture medium, more than 80% of B10 cells differentiated into lipid-laden cells that stained with oil-red. After 14 days of culture in osteogenic medium, B10 cells differentiated into osteoblasts, which were confirmed by strong alkaline phosphatase staining. Differentiation of B10 cells into chondroblasts was determined by staining the cell pellets with Alcian blue which identifies proteoglycan extracellular matrix, specific components of cartilage tissues (Figures 2B–D). Differentiation of B10 cells into adipocytes, osteoblasts and chondroblasts was also confirmed by RT-PCR analysis (Figures 2B–D). As in primary human MSCs [3], [35], differentiated B10 cells expressed markers for adipocytes, osteoblasts or chondrocytes following culture under the appropriate differentiation-inducing conditions [36].

### Genetic expression of primary human MSCs and B10 cells

**Figure 3:**
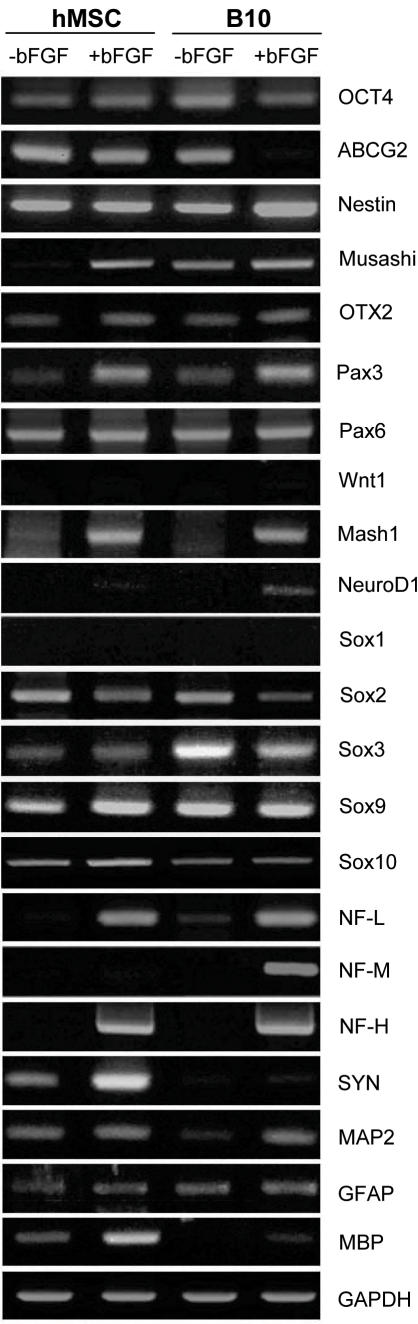
Neuronal phenotype and neuronal differentiation of B10 cells. Gene expression of stem cell markers and neuronal markers was examined by RT-PCR. B10 cells reproduce genetic features of primary human bone marrow MSCs by expressing ABCG2 and Oct-4 as general stem cell markers, Mash 1 and Otx 2 as neurogenic bHLH genes, nestin as a cell type specific marker for neural stem cells, and low-molecular weight neurofilament protein (NF-L), intermediate molecular weight NF (NF-M) and high molecular weight NF (NF-H) as specific markers for neurons. B10 cells were grown in medium containing basic FGF for 2 weeks, In these conditions, B10 cells were demonstrated to differentiate into neurons.

We examined expression of the genes involved in neural differentiation of primary human MSCs and B10 cells (Figure 3A). Both primary MSCs and B10 cells express Oct-4, which is a cell type-specific marker for the pluripotent stem cells [37] and B10 cells expressed ABCG2 [38] as a novel stem cell marker, nestin [39] and Musashi1 [40], both cell type-specific markers for neural stem cells before and after bFGF treatment (Figure 3).

The bHLH transcription factors investigated in neuronal differentiation included Otx2, Pax3, Pax6, Wnt1, Hash1 and NeuroD1. Expression levels of Pax3, which is involved in the brain regionalizaion and establishment of dorso-ventral polarity of the spinal cord during early neurogenesis [41] were increased in both primary human MSCs and B10 cells following bFGF treatment of 2 weeks. Wnt1 expression [42] in both primary MSCs and B10 cells increased markedly after bFGF treatment. Expression of Mash1 [43], [44], Otx-2 [45] and Pax6 [46], other transcription factors crucial during the early phase of neurogenesis was demonstrated in control primary human MSCs and B10 cells, and expression levels of these transcription factors stayed steady after bFGF treatment. Expression of NeuroD1, which is expressed in subsets of CNS neurons during terminal differentiation[47], was demonstrated after induction by bFGF, indicating that the transcription factor NeuroD1 participates closely in trans-differentiation of MSCs into neurons. Additionally, we investigated expression of Sox proteins, which are important in cell fate determination of progenitor cells if they become neurons or glial cells [48]. Sox1, Sox2 or Sox3 maintain a stem cell-like state [49], [50], and Sox9 alters the potential of stem cells from neurogenic to gliogenic [51], whereas Sox10 is essential for terminal oligodendrocyte differentiation [52]. Expression levels of Sox2 and Sox3 decreased in especially B10 cells following bFGF treatment of 2 weeks. Low molecular weight neurofilament protein (NF-L), found in young immature neurons, increased considerably after 2 weeks of bFGF treatment and expression of mature NF forms, NF-M and NF-H, appeared after bFGF treatment. Genetical analysis also showed increased expression of additional neuronal cell type-specific markers, synaptophysin and MAP2. Expression of cell type-specific marker for glial cells, GFAP for astrocytes, was found in primary human MSCs and B10 cells regardless of the status of bFGF treatment. Expression of MBP for oligodendrocytes was found in control primary MSCs but an increase in its expression was found following the bFGF treatment, and in B10 cells MBP expression was demonstrated only after the bFGF treatment. Both primary MSCs and B10 cells expressed ABCG2 [38] as a novel stem cell marker, nestin [39] and Musashi1 [40] both cell type-specific markers for neural stem cells (Figure 3A) before and after bFGF treatment.

### Neural differentiation of B10 cells in vitro

**Figure 4:**
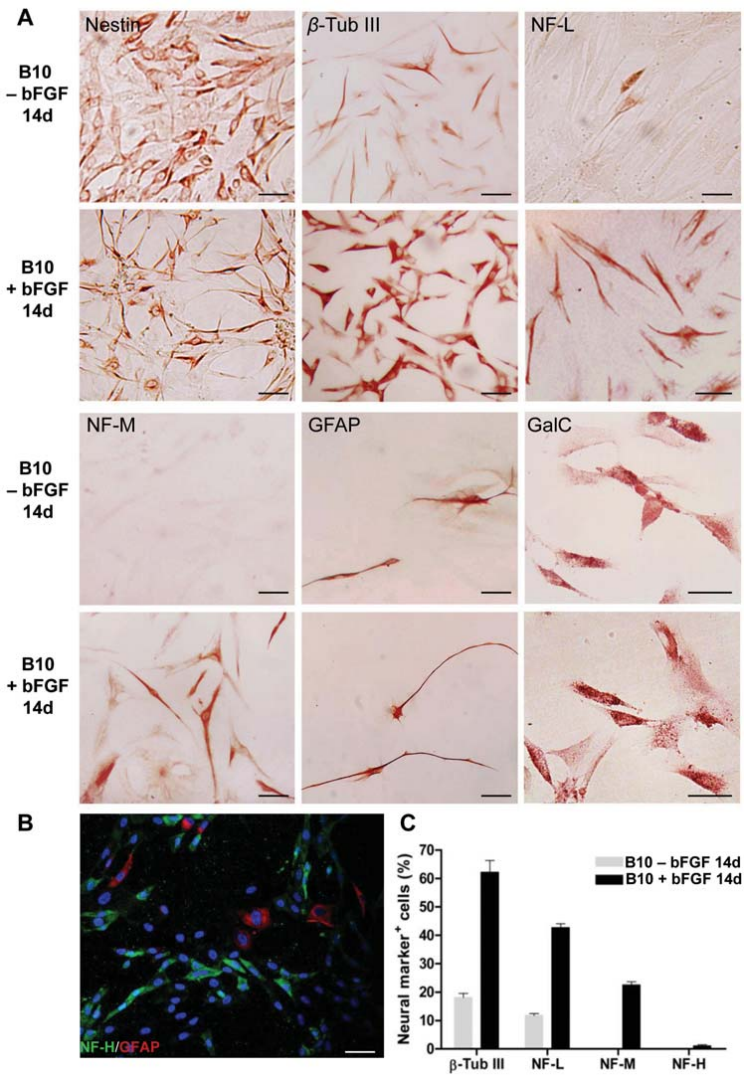
Neuronal phenotype and neuronal differentiation of B10 cells. (A): B10 cells in culture were induced to differentiate into neurons in serum-containing medium supplemented with 100 ng/ml basic FGF for 2 weeks and then processed for nestin (for neural stem cells), β-tubulin III, NF-L, NF-M and NF-H (for neurons), GFAP (for astrocytes) and galactocerebroside (for oligodendrocytes). Bar inciates 20 µm. (B) Two color Immunofluorescence microscopy demonstarating pluripotential differentiation of B10 cells into neurons (as shown by NF-L/green staining) and astrocytes (GFAP/red). B10 cells were grown in serum-containing medium supplemented with basic FGF for 2 weeks. (C) Percentage of neural cells immunoreaction-positive for β-tubulin III, NF-L, NF-M and NF-H is determined in the B10 cells treated with 100 ng/ml bFGF and the controls (in the absence of bFGF). Cell number was determined in 5 fields of low magnification. Error bars = SEM.

Less than 20% of B10 cells was immunoreactive for neuronal markers, β–tubulin III, NF-L or NF-M when they were grown in regular feeding medium in the absence of bFGF supplementation, while treatment with bFGF for 2 weeks, most of B10 cells showed bipolar or multipolar morphology with branched processes, and the proportion of B10 cells expressing cell type sepcific markers increases considerably (Fig. 4A). B10 cells expressing β-tubulin III increased from 18.1±1.4% in the control to 62.2±4.1%, NF-L from 11.8±0.6% to 42.8±1.3%, NF-M from 0 to 22.6±1.1% and NF-H from 0 to 1.2±0.3% (Figure 4C). Less than 2–4% of all B10 cells differentiated into GFAP-expressing astrocytes *in vitro* (Figure 4A). Following treatment with bFGF, a larger number of NF-positive neurons were found in cultures as compared with that of GFAP-positive astrocytes (Fig. 4B). In the RT-PCR analysis, B10 cells expressed MBP, a cell type specific marker for oligodendrocytes (Fig. 3A), and in immunocytochemical investigation a small number of B10 cells expressing oligodendrocyte specific markers such as galactocerebroside was found in culture (Fig. 4A). These results indicate that bFGF is a highly effective modulator/inducer of neuronal differentiation from the undifferentiated and uncommitted MSCs.

### Generation of sodium current in B10 cells after basic FGF treatment

**Figure 5:**
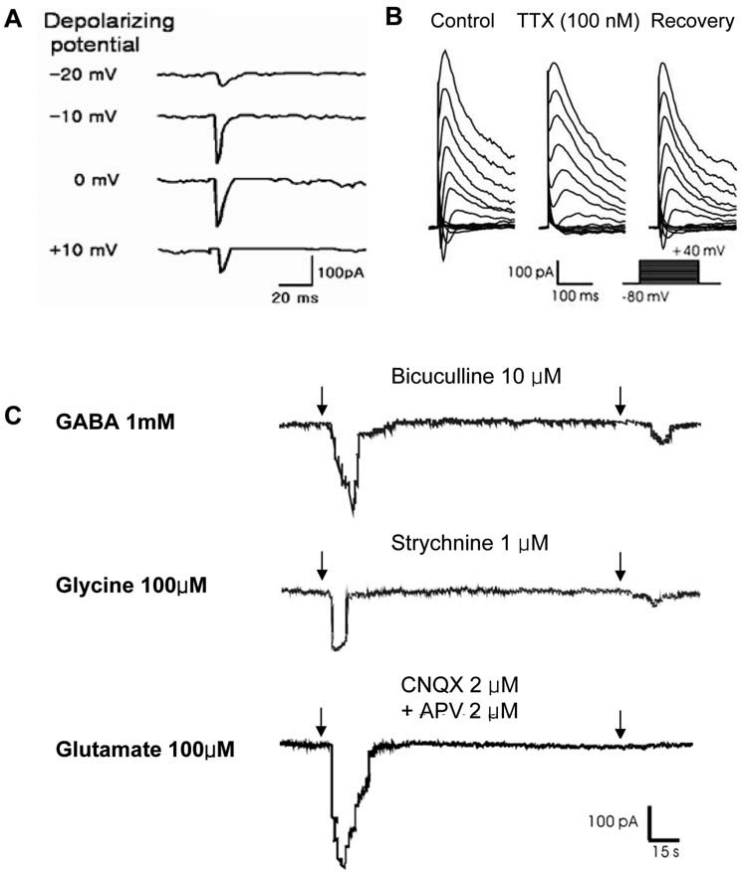
Electrophysiology of B10 cells demonstrating neuronal characteristics. (A): Voltage-gated channel activation in B10 cells following treatment with 100 ng/ml bFGF for 11–14 days. The holding potential was −80 mV and depolarizing steps were applied from −80 mV to +40 mV in 10 mV increments. (B): Na+ currents were activated from a depolarizing step of −40 mV and blocked reversibly by 100 nM tetrodotoxin (TTX). B10 cells grown with bFGF showed sustained outward currents as well. These currents showed a voltage-dependence and kinetics characteristics for delayed rectifier K+ channels. (C): Neurotransmitter-elicited currents. Application of 1 mM GABA, 100 µM glycine, or 100 µM glutamate resulted in inward currents in the presence or absence of specific antagonists, bicuculline (10 µM), strychnine (1 µM) or APV (200 µM), respectively.

Electrophysiological recordings from B10 MSCs treated with bFGF for 11–14 days were examined with whole-cell voltage clamp (Figure 5). More than 50% (11 of 20 cells examined) of B10 cells grown for 11–14 days in the presence of bFGF expressed sodium currents (Figure 5A), while control B10 cells grown in the absence of bFGF were quiescent and exhibited virtually no sodium current (n = 12). In addition, the sodium currents were blocked by100 nM tetrodotoxin, and then sodium current reappeared upon washing out tetrodotoxin (Figure 5B). Concomitant with sodium current induction, B10 cells grown with bFGF showed sustained outward currents of a several hundred pA up to 1 nA. These currents showed a voltage-dependence and kinetics characteristics for delayed rectifier K+ channels. Positive responses of whole-cell voltage-clamped B10 cells to neurotransmitters (1 mM GABA, 100 µM glycine, and 100 µM N-methyl-D-aspartate) were subsequently recorded under −80 mV of holding potential (n = 6 for each neurotransmitters) (Figure 5C). All neurotransmitters evoked inward currents in more than 50% of B10 cells, and neurotransmitter-elicited currents were reversibly blocked by specific antagonists of these receptors: bicuculline (10 µM), strychnine (1 µM), and mix of APV (2 µM) and CNQX (2 µM), respectively. The neurotransmitter-evoked currents were mediated by their respective ionotropic receptors- GABA_A_ receptor, glycine receptor, and NMDA/AMPA/KA receptors.

### B10 cells migrate to hemorrhagic lesion, corpus callosum and hippocampus in ICH model

**Figure 6:**
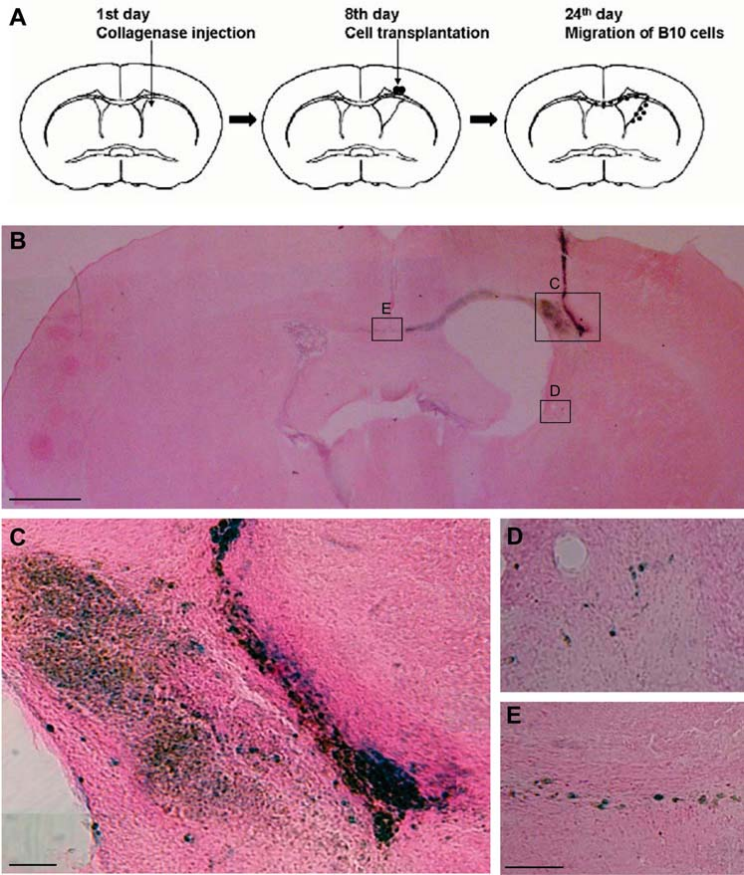
Survival of B10 cells transplanted on the area overlying intracerebral hemorrhage lesion in adult mouse brain. (A): Eight days after lesioning by injection of collagenase, B10 cells were transplanted. Two weeks after transplantation, an extensive migration of B10 cells was identified. (B): Two weeks post-operation, LacZ-positive B10 cells were noted migrating away from the injection track and located along the hemorrhage borders and also into contralateral hemisphere via corpus callosum. (C)–(E): Higher magnification of areas identified in (B) indicating a good survival and long-distance migration of LacZ-positive B10 cells from the injection site. Bar in (B) indicates 0.5 mm, and bars in (C)–(E) are 100 µm.

Expansion of lateral ventricle by fusion with hematoma cavity appeared 2–3 weeks after ICH stroke. Primary MSCs and B10 cells labeled with adenovirus-LacZ were transplanted in the cortex overlying hemorrhage core along the anterior-posterior axis of the ipsilateral striatum 7 days after ICH stroke (Figure 6A). At 2 weeks post-transplantation, brain sections were processed for β-gal staining to identify the transplanted cells and injection tracts of the transplants were clearly visible and most of the LacZ-positive B10 cells migrated from the original injection sites and localized at the boundary of lesion cavity which was fused with dilated lateral ventricles (Figures 6B–E). In addition, a large number of LacZ-positive B10 cells was found in the corpus callosum migrating toward contralateral hemisphere (Figures 6B and 6E). Identical results were obtained with transplantation studies with primary human MSCs in ICH rats (data not shown).

### Transplanted B10 cells in ICH brain express neural cell-specific markers

**Figure 7:**
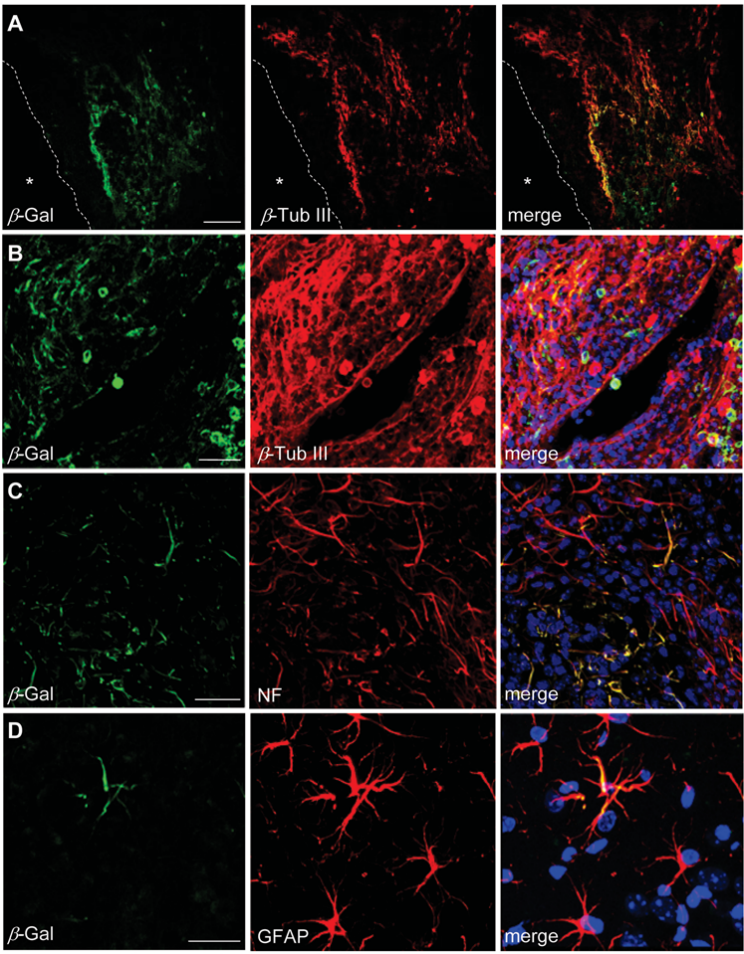
B10 cells labeled by β-galactosidase (β-gal) transplanted into the cortex overlying the intracerebral hemorrhage (ICH) lesion are found to differentiate into neurons and astrocytes. Two weeks post-transplantation at the lesion sites. In vivo differentiation of β-gal-positive B10 cells into neurons was shown by β-tubulin III (A and B), and neurofilament (C) and into astrocytes as shown by GFAP (D) staining. Bar indicates 50 µm.

**Figure 8:**
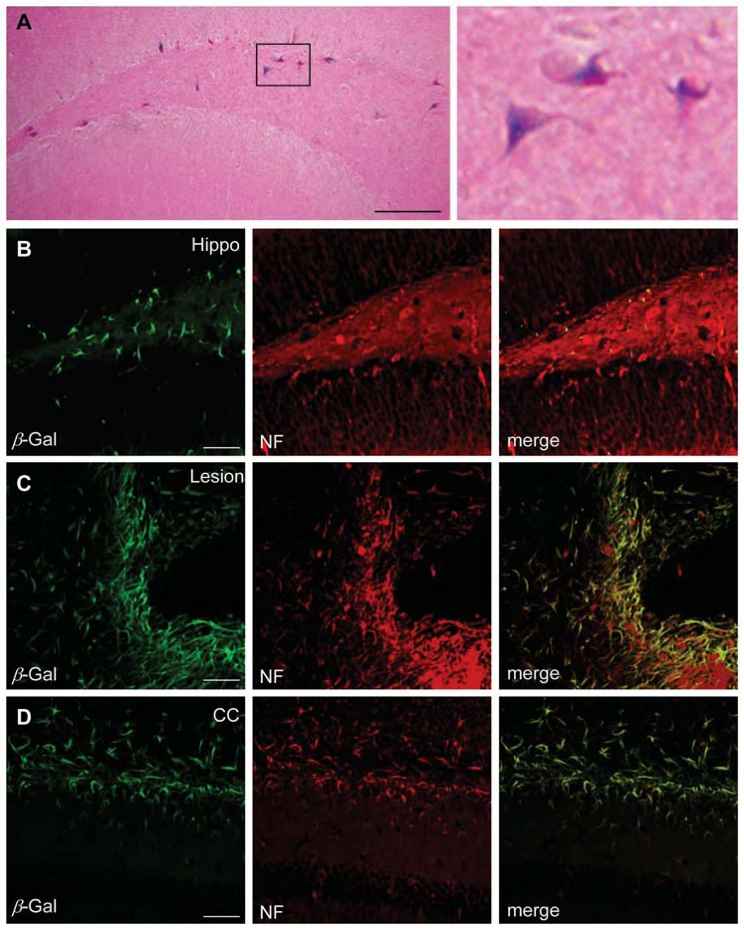
B10 cells labeled by β-gal differentiate into neurofilament-positive neurons in the striatal hemorrhage lesion site but also in corpus callosum (CC) and hippocampus indicating that the B10 cells are capable of an extensive migration. Six weeks post-transplantation. (A): LacZ (β-gal)-positive B10 cells are found migrating into the pyramidal cell layer of hippocampus. Bar indicates 100 µm. (B)–(D): β-galactosidase-positive B10 cells differentiate into neurofilament (NF)-positive neurons in hippocampus in the lesion side (B), in the boundary of striatal lesion (C) and corpus callosum (D) at 6 weeks post-transplantation. Bar indicates 100 µm.

Two weeks after ipsilateral cortical transplantion, a large number of B10 cells surrounding the hemorrhage core expressed neuronal markers, β-tubulin III and neurofilament proteins (NF) (Figures 7A–C) and a smaller number of β-gal-positive B10 cells expressed GFAP, a specific marker for astrocytes (Figure 7D). We also found β-gal-positive B10 cells to the other brain sites including corpus callosum and hippocampus at pyramidal layer of CA2 and CA3 (Figure 8A). At 6 weeks post-transplantation, the majority of β-gal-positive B10 cells located in the lesion sites or vicinity expressed NF indicating that a major population of grafted B10 cells differentiates into neurons with elapsed time of 3–4 weeks (Figures 8B–D). None of β-gal-positive B10 cells was found to express CNPase immunoreaction, a specific cell type marker for oligodendrocytes (data not shown). At two weeks and 6 weeks post-transplantation, a considerable number of LacZ-positive/β-gal-positive B10 cells were found in corpus callosum and hippocampus mostly expressing neurofilament phenotype.

### Recovery of behavioral deficits in ICH model by B10 cell transplantation

**Figure 9:**
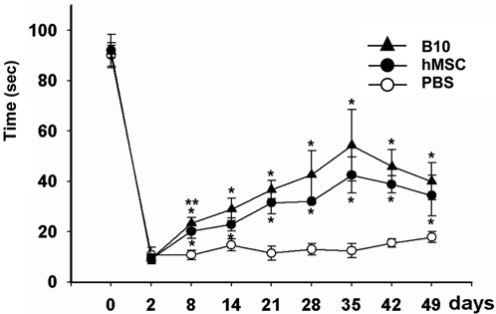
Funtional recovery of ICH mice following B10 cell transplantation as shown by rotarod test. Fuctional improvement in mouse ICH model following transplantation of clonal B10 cells, primary human MSCs and PBS control as determined by rotarod test. Error bar = SEM. *p<0.05 compared to PBS control. **p<0.05 between B10 cells and primary human MSCs.

Motor performance of ICH animals was determined by the rotarod test in animals receiving PBS, animals transplanted with primary human MSCs or B10 cells (Figure 9). There is a highly significant difference between B10-injected and PBS-injected mice in two-way ANOVA (F = 50.32, P<0.0001). While no behavioral recovery was noted in PBS injected control animals, ICH mice grafted with B10 cells showed a significant improvement in motor performance from 8 days post-transplantation and the improvement lasted for up to 7 weeks post-transplantation as compared with controls by one-way ANOVA (8 days p = 0.041, 14 days p = 0.004, 21 days p = 0.002, 28 days p = 0.007, 35 days p = 0.026, 42 days p = 0.001, 49 days p = 0.040). Behavioral improvement was also noted in ICH mice grafted with primary human MSCs (8 days p = 0.005, 14 days p = 0.022, 21 days p = 0.001, 28 days p = 0.040, 35 days p = 0.046, 42 days p = 0.012, 49 days p = 0.011). Significant difference of recovery by transplantation was detected between human MSCs and B10 cells (p = 0.016) only on 8 days post-transplantation.

### B10 cells produce NGF and BDNF in vitro and in vivo

**Figure 10:**
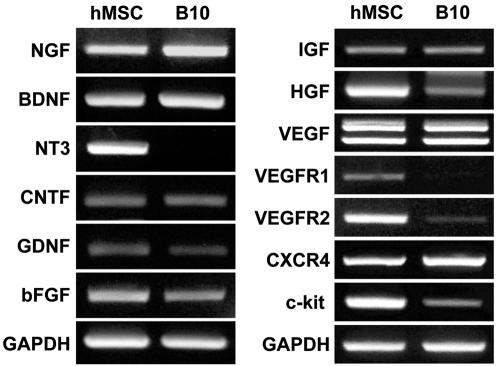
Expression of neurotrophic factors in B10 cells by RT-PCR Gene expression of neurotrophic factors and cytokine receptors (involved in cellular migration of B10 cells) was analyzed by RT-PCR in primary human MSCs and B10 cells. B10 cells express genes for neurotrophic factors including NGF, BDNF, CNTF, GDNF, bFGF, IGF, HGF and VEGF. In addition, receptors for cytokines known for induction of cell migration, VEGFR1, CXCR4 and c-kit are expressed by B10 cells.

**Figure 11:**
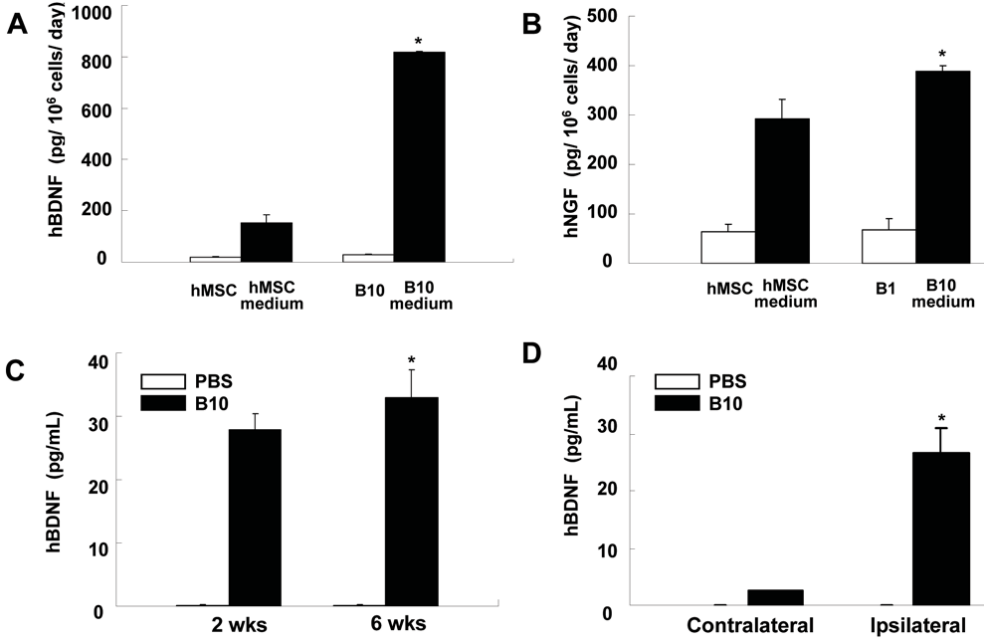
Expression of neurotrophic factors in B10 cells in vitro and in vivo. (A): ELISA assay for human BDNF demonstrates that B10 cells secrete BDNF into culture medium approximately 5 times over primary MSCs. Error bar = SEM. *p<0.001 (B): ELISA assay for human NGF showed that both B10 cells and primary MSCs secrete substantial amount of NGF into culture medium. Error bar = SEM. *p<0.01. (C) Levels of human BDNF in ipsilateral sections (transplanted side) of B10 cell transplanted Brain. *p<0.0001) as compared to those of PBS injected brain (undetectable) at 2, 4 and 6 weeks post-transplantation. (D) Levels of human BDNF in ipsilateral brain sections of B10 cell transplanted side as compared to contralateral sections without transplanted B10 cells at 2, 4 and 6 weeks post-transplantation. In the PBS injected control brain, BDNF level was undetectable. *p<0.01.

Production of cytokines and growth factors by B10 cells was determined by RT-PCR. As in primary human MSCs, B10 cells express high levels of NGF, BDNF, CNTF, GDNF, bFGF, IGF-I, HGF and VEGF (Figure 10). It is interesting to note that the message for NT3 demonstrated in primary MSCs is not detected in B10 cells. In addition to growth factors, the RT-PCR study has demonstrated that both primary human MSCs and B10 cells express mRNA for VEGFR, CXCR4 and c-kit, cellular receptors known for close involvement in cellular migration (Figure 10). These results indicate that the pathways involving SCF/c-kit, SDF-1/CXCR4 and VEGF/VEGFR are important in the migration of MSCs to the sites of ICH brain damage and also to corpus callosum and hippocampus. ELISA analyses indicated that the levels of human BDNF released by B10 cells *in vitro* were 8.18±0.04 ng/10^6^ cells/day (mean±SEM) in the spent medium of B10 culture of 24 hr, which were five times higher than those from human primary MSCs (1.53±0.31 ng/10^6^ cells/day, p<0.001) (Figure 11A). In addition to BDNF, levels of human NGF were determined by an ELISA kit specific for human NGF in the culture supernatants of B10 cells and primary MSCs (Figure 11B). The levels of NGF are very high in both B10 cells and primary MSCs (388.1±6.6 vs 292.0±23.2 pg/10^6^ cells/day, p<0.01). B10 cells secrete 40% higher level of NGF into the culture medium.

We also examined the levels of human BDNF in ICH brain sections 2, 4 and 6 weeks post-transplantation (Figure 11C). BDNF levels at 2, 4 and 6 weeks post-transplantation in the brain sections of B10-transplanted loci were 9.25±1.00 pg/mg protein (2 wks), 13.12±0.67 pg/mg protein (4 wks), and 8.63±0.63 pg/mg protein (6 wks), while production of human BDNF in the brain sections of PBS-injected mice was below the detectable level of the ELISA assay. Increased BDNF levels found in the transplanted ICH brain sections of 6 weeks post-operation indicate that a large number of implanted B10 cells survived and released substantial amount of human BDNF even after 6 weeks of transplantation.

Next we examined the BDNF levels in ipsilateral (graft side) and contralateral sides (nongraft side) of brain sections from both B10-transplanted and PBS-injected mice (Figure 11D). BDNF levels in the ipsilateral (B10 graft side) brain sections were 9.25±1.00 pg/mg protein at 2 weeks, 13.12±0.67 pg/mg protein at 4 weeks and 8.63±0.63 pg/ml at 6 weeks. BDNF levels in the contralateral side (non-grafted side) of brain sections registered as 1.00±0.04 pg/mg protein at 2 weeks, 2.59±0.30 pg/mg protein at 4 weeks and 1.94±0.08 pg/ml at 6 weeks. Low levels of human BDNF detected in the contralateral sides of the brain are attributed to a small number of β-gal/BDNF double-positive B10 cells which migrate to the opposite side of the brain via corpus callosum (see Figure 6). BDNF levels in both ipsilateral and contralateral brain sections of PBS-injected mice were below the detectable level of the ELISA assay. In support of B10 cells' capacity to produce BDNF in high levels in the brain, a large number of transplanted B10 cells showing β-gal/BDNF-double positive reaction were found in the lesion sites. Smaller number of BDNF-expressing B10 cells was also found in corpus callosum and hippocampus (data not shown).

### Survival of transplanted B10 cells in vivo

At 7 days after ICH, 2×10^5^ B10 cells in a total fluid volume of 2 µl were transplanted into ipsilateral cortex, 2 mm cranial to the hemorrhagic lesion. Total number of LacZ-positive B10 cells in the brain sections from ICH animals was determined by stereological analysis at 2 weeks and 6-weeks post-transplantation. The results indicate that cell survival at 2 weeks post-transplatation is 83,100±1140 cells (41.6±1.7% of the initial population of 200,000 cells) and at 6 weeks the number is 40340±2850 cells (20.2±2.5% of the initial population of 200,000 cells). Although number of B10 cells transplanted was 200,000, the cell count of live cells immediately prior to transplantation (by trypan blue exclusion test) indicated that actual number of live cells was 80% (160,000 cells). It is necessary to remove cells from culture dishes by trypsin treatment for the preparation of cells for transplantation and this procedure damages cells. From this figure, the results of cell survival of grafted B10 human MSCs in the brain of ICH animals could be 51.9% at 2 weeks post-transplantation and 25.2% at 6 weeks. Further study is necessary to investigate a long-term follow-up for the period of 6 months to one year to determine the fate of grafted cells and such a study is underway in our laboratory.

## Discussion

In the present study, we report the generation of permanent, stable human mesenchymal stem cell (MSC) lines with properties of self-renewal and pluripotency. B10 immortalized human MSC line is capable of differentiation into fat, bone and cartilage and also into neurons and glial cells *in vitro* and *in vivo*. Following transplantation into the brain of mouse intracerebral hemorrhage stroke (ICH) model, B10 cells survive, differentiate into neurons and astrocytes and induce functional improvement in these animals. B10 human MSC line was established by transfecting primary cultures of human bone marrow MSCs with a retroviral vector encoding v-myc. The phenotypic expression of B10 is consistent over culture passages and is in accordance with the phenotypes of primary human MSCs as previously reported [3], [35], [53]. Thus B10 cells express MSC-specific cell type markers including CD13, CD29 (integrin-β1), CD44 (endoglin), CD49b, CD90 (Thy-1) and CD166 (Figure 2A and Table 1).

The present study in immortalization and cloning of human MSCs into stable permanent cell lines represent our attempt to overcome some of the limitations of primary cultures of MSCs and provide a potentially significant experimental model for biomedical research. We have experience of growing primary human MSCs isolated from fetal bone marrow for more than 3 years and found out that the primary human MSCs do not remain in a continuous proliferative state in serum free conditions (with addition of bFGF and EGF), and after a finite number of mitoses (up to 20), they cease to divide and differentiate. In order to maintain them in a proliferative state, we have transduced primary human MSCs with the retroviral vector encoding immortalizing gene v-*myc*. Stable immortalized cell lines of human MSC as described in the present study should provide unlimited number of homogenous cells derived from a single stem cell and facilitate follow up of progeny of the same stem cell for prolonged period, over many generations. When differences in behaviors are observed for different stem cells in different miniwells, one cannot discern whether these represent stochastic changes, or are the product of subtle differences in microenvironmental signals, or reflect a fundamental heterogeneity of the stem cell population. Immortalized stem cell line derived from a single cell can circumvent these problems and produces a clear-cut outcome.

There is a concern about the use of oncogenes for generation of immortalized stem cell lines, as the oncogene in question might cause tumor/ectopic formation if cells proliferate indefinitely in the brain over time following transplantation. Although we did not find tumor formation *in vivo* in animals following brain transplantation of B10 human MSCs in the present study, we do not plan or project clinical trials in patients using the B10 human MSCs. There is, however, an alternative approach to circumvent this concern. We have recently generated immortalized human MSC lines by the conditional expression of v-*myc* under control of a regulatable tetracycline (Tet) promoter. Addition of Tet to the medium activates the v-*myc* protein allowing the clonally isolated human MSCs to proliferate rapidly while holding differentiation essentially in abeyance; in the absence of Tet, the human MSCs cease proliferating and proceed to differentiate into various cell types (Manuscript in preparation).

Previous works have demonstrated that human MSCs express genes characteristic of multilineage cell type prior to unilineage commitment [54], [55]. Rodent marrow derived-multipotent progenitor cells express Oct-4 as a necessary marker for pluripotency in stem cells [56] and Otx1/2 expressed at early stages of neuroectoderm [1]. Human MSCs also express Oct-4 [6] and nestin [57] as neural stem cell markers and even β-tubulin III, a neurons specific marker and GFAP, a marker for astrocytes, were expressed prior to undergoing differentiation [7]. Moreover, proneural genes, Otx-1, Neurogenin 2 and Musashi 1 as well as Oct-4 and nestin have been expressed in mRNA level in human MSCs as reorted earlier in adult derived human MSCs [6]. In the present study, B10 as a clonal cell line originated from a single human MSC, expresses Oct-4 and ABCG2 as general markers for stem cells, nestin as a neural stem cell marker, and Mash-1 and Otx-2 which are basic helix-loop-helix (bHLH) transcription factors important in developmental process of neurogenesis. Thus B10 human MSC cell line has same differentiation capacity into human neural stem cells as in primary human MSCs.

Previous studies have reported that the MSCs derived from bone marrow differentiate into neuronal lineage cells [56], [57] and expression of β-tubulin III in early phase of neuronal differentiation was gradually replaced by neurofilament (NF) proteins as human MSCs become more mature neuronal lineage cells [58], [59]. Increased expression of β-tubulin III and NF-L in MSCs following bFGF treatment as shown in the present study suggests that B10 differentiate into neuronal progenitor cells and neurons via bFGF-mediated differentiation pathway. Moreover, B10 cells expressing NF proteins were identified to generate inward currents of voltage-activated Na+ channels, which indicate that the neuronally differentiated B10 cells have electrophysiological characteristics of mature neurons. Further study is necessary to characterize morphological and functional phenotypes in these cells such as expression of neurotransmitter type and formation of synaptic connection.

MSCs are known to show a capacity to migrate and differentiate into various cell types, and to be integrated into the host tissues following *in vivo* transplantation. Following bone marrow transplantation, donor-derived cells have been found in multiple non-hematopoietic tissues, including liver and the brain. In the brain, grafted marrow-derived stem cells could express the neural antigens Neu-N and nestin [15], [60], [61]. Previous studies have shown that bone marrow MSCs injected intravenously or transplanted in cerebrum significantly reduced motor and neurological deficits in animal models of traumatic brain injury [62], Parkinson disease [63] and brain ischemia [13]–[15].

In the present study, transplantation of B10 MSCs into the ipsilateral cortex above the intracerebral hemorrhagic lesion showed that a large number of B10 cells survive, migrate along the boundary zones adjacent to the hemorrhage core and differentiate into neurons and astrocytes. These observations are consistent with the findings of previous studies that the grafted bone marrow stem cells could differentiate into neural cells in the microenvironment of lesioned brain [13], [15]. It is evident that the neurogenesis and migration of transplanted MSCs are influenced by the cues or signals in the microenvironment of damaged brain. Previous studies have shown that a limited degree of endogeneous neurogenesis occur in damaged region, which was induced by stroke [64], [65]. Further studies are needed to determine whether grafted B10 MSCs could integrate among the neurons of host brain, make new synaptic connections with them and replace lost neurons. Electrophysiological studies undergoing in our laboratory in brain slices obtained from rat brain grafted with B10 cells so far did not yield positive evidence of new synaptic connection.

A considerable number of the transplanted B10 migrate to the boundary region, corpus callosum and hippocampus in the contralateral side of transplantation and differentiate into neurons even in far beyond hemorrhagic stroke site. In a previous study, human neural progenitor cells integrate into hippocampus and corpus callosum following transplantation in lateral ventricle and striatum of neonatal and adult animals [66]. Transplanted MSCs migrated along corpus callosum [15], [67] and to hippocampus [14], [68] in normal rats or rats with ischemic lesions. Although mechanism by which MSCs and neural stem cells migrate extensively in selective manner to the pathological lesions is unclear, it is suggested that the transplanted B10 cells mimic the behavior of neural stem cells that are recruited by chemoattactant signals produced at CNS injury sites such as cytokines including stem cell factor (SCF) [69], stromal cell derived factor-1 (SDF-1) [70] and vascular endothelial cell growth factor (VEGF) [71]. Other cytokines with important functions in CNS development including basic FGF, EGF and TGFα are also shown to increase ischemia-induced proliferation and migration of neural progenitor cells [72]. Since B10 cells express c-kit, the receptor for SCF, CXCR4, the receptor for SDF-1, and VEGFR1, receptor for VEGF (Figure 10), pathways involving SCF/c-kit, SDF-1/CXCR4 and VEGF/VEGFR are involved in the migration of MSCs to the sites of ICH brain damage and also to corpus callosum and hippocampus. Migration of MSCs toward sites of brain injury may represent an adaptive response of MSCs for the purpose of limiting tissue injury or repair the tissue damage. The mechanism by which the B10 MSCs undergo selective and long distance migration to non-injured sites of corpus callosum and hippocampus might differ from that for the ICH injury and further studies are required to identify the signal(s) for the MSC migration to apparently normal brain region.

Brain microenvironment is important in determining survival, migration and differentiation of exogenously transplanted progenitor cells and stem cells. Following the collagenase injection into the striatum, a profuse hemorrhage in the area caused by blood vessels damaged by the proteinase enzyme ensues and the hemorrhage core routinely is absorbed within a week or two, but immune cells released from the vessels remain in the hemorrhage core area. Transplantation of B10 human MSCs is conducted one week after the hemorrhage lesion, thus host immune cells might attack the newly implanted human MSCs in the area. On the other hand, it is known that damaged brain cells and tissues of the host are also capable of releasing molecules that stimulate production of neurotrophic factors in transplanted MSCs [73].

In the present study, no immunosuppressant such as cyclosporine A was utilized to inhibit immune reaction and promote the long-term survival of implanted MSCs in the ICH animals. In earlier studies we have intravenously transplanted immortalized stable human neural stem cells in ICH and focal ischemia model rats without administering immunosuppressant, and found a good survival of grafted NSCs in the brain and a good functional recovery in these animals [21]–[24]. However, low survival rate of grafted B10 cells in ICH mice as demonstrated in the present study is a grave concern. The survival rate of transplanted B10 cells at 2 weeks post-transplantation is 41% (52% with correction) and that of 6 weeks is 20% (25% with correction). Obvious cause for such poor survival rate of human MSCs in experimental animals is immune-mediated mechanisms by which grafted cells were attacked and destroyed. For that reason, we have to employ immunosuppressants in our future studies to protect grafted human MSCs from cell death in experimental animals. It should be noted that the immunosuppressant cyclosporine has recently been demonstrated to exert neuroprotection in experimental stroke among other disease models [74]–[76].

The importance of immunosuppresion in the event of clinical trials using human MSCs in patients suffering from stroke or other neurological diseases is well recognized. Immunological rejection of neural transplants poses a significant problem to be overcome in order to conduct successfully stem cell-based cell therapy in human patients. A previous study has suggested that the use of progenitors and stem cells for neural grafting is more promising, as these could be maintained *in vitro* until use, and evoke less immunogenic responses when compared to primary grafts; implantation of immortalized mouse neural stem cells in rat ischemia model has resulted in a good survival at 2 weeks post-transplantation in the absence of immune reaction caused by grafted cells [77]. Further investigations into the specific mechanisms underlying drug actions of immunosuppressants in experimental stroke will certainly improve the therapeutic potential of these drugs for stem cell-based cell therapy.

Endogeneous or exogenously injected neurotrophic factors including BDNF, GDNF, CNTF and NT3 had neuroprotective effect in damaged brain including stroke [78]–[80]. Extracts from ischemic rat brain have induced the production of BDNF, bFGF, VEGF and HGF in human MSCs in culture [73]. In addition, mouse MSCs are shown to express VEGFα and EGF in cDNA microarray analysis [81]. A recent study has also reported that transplanted porcine choroids plexus in microcapsules improved behavioral performance and decreased the pathological lesion size in rat ischemia stroke model by producing several trophic factors including NGF, BDNF and GDNF [82].

Our RT-PCR studies show that B10 human MSCs as in primary human MSCs express BDNF, GDNF, CNTF, bFGF, VEGF, HGF and IGF that may work as neuroprotective factors in the ICH mice. B10 MSCs secrete both NGF and BDNF proteins in higher concentrations *in vitro* and *in vivo* than primary human MSCs. Recent studies have shown that the intracerebral injection of MSCs transfected with the BDNF or GDNF gene resulted in improved function and reduced ischemic damage in a rat stroke model of middle cerebral artery occlusion [83]. In the present study, grafted B10 cells located in peri-hemorrhagic lesion sites express strong BDNF activity, which indicate that the grafted B10 cells secrete BDNF neurotrophic factor in the microenvironment of ICH and promote survival of host neurons and functional recovery of ICH animals.

As for the parameters in evaluation of treatment efficacy in stem cell-based cell therapy for stoke animal models, several parameters such as improvement in behavior, number of cells differentiating into neurons, degree of cellular migration and number of surviving cells in the graft, could be considered. From the results described in the present study and our previous studies in animal models of stroke with transplantation of immortalized human neural stem cells [20]–[24], two parameters, behavioral recovery and survival of grafted cells, have paramount importance. Without behavioral improvement following cell therapy with stem cells, the study in question is a total failure, and no behavioral improvement could be expected in the absence of good survival in grafted cells.

In conclusion, B10 human MSC cell line can be induced to differentiate mostly into neurons and smaller number of astrocytes *in vitro* and *in vivo* and has a potential to produce a number of neuroprotective factors including NGF and BDNF. The present study demonstrates that B10 human MSC cell line is not only a useful tool for the studies of organogenesis and specifically for the neurogenesis, but also as a renewable cell source for cell therapy studies in animal models of stroke and other neurological disorders.
